# Screening of a *Brassica napus* bacterial artificial chromosome library using highly parallel single nucleotide polymorphism assays

**DOI:** 10.1186/1471-2164-14-603

**Published:** 2013-09-06

**Authors:** Hieu Xuan Cao, Renate Schmidt

**Affiliations:** 1 Leibniz Institute of Plant Genetics and Crop Plant Research (IPK), Corrensstraße 3, OT Gatersleben, D-06466 Stadt Seeland, Germany

**Keywords:** BAC library, *Brassica napus*, Genotyping, Multidimensional pools, PCR screening, Polyploidy, SNP

## Abstract

**Background:**

Efficient screening of bacterial artificial chromosome (BAC) libraries with polymerase chain reaction (PCR)-based markers is feasible provided that a multidimensional pooling strategy is implemented. Single nucleotide polymorphisms (SNPs) can be screened in multiplexed format, therefore this marker type lends itself particularly well for medium- to high-throughput applications. Combining the power of multiplex-PCR assays with a multidimensional pooling system may prove to be especially challenging in a polyploid genome. In polyploid genomes two classes of SNPs need to be distinguished, polymorphisms between accessions (intragenomic SNPs) and those differentiating between homoeologous genomes (intergenomic SNPs). We have assessed whether the highly parallel Illumina GoldenGate® Genotyping Assay is suitable for the screening of a BAC library of the polyploid *Brassica napus* genome.

**Results:**

A multidimensional screening platform was developed for a *Brassica napus* BAC library which is composed of almost 83,000 clones. Intragenomic and intergenomic SNPs were included in Illumina’s GoldenGate® Genotyping Assay and both SNP classes were used successfully for screening of the multidimensional BAC pools of the *Brassica napus* library. An optimized scoring method is proposed which is especially valuable for SNP calling of intergenomic SNPs. Validation of the genotyping results by independent methods revealed a success of approximately 80% for the multiplex PCR-based screening regardless of whether intra- or intergenomic SNPs were evaluated.

**Conclusions:**

Illumina’s GoldenGate® Genotyping Assay can be efficiently used for screening of multidimensional *Brassica napus* BAC pools. SNP calling was specifically tailored for the evaluation of BAC pool screening data. The developed scoring method can be implemented independently of plant reference samples. It is demonstrated that intergenomic SNPs represent a powerful tool for BAC library screening of a polyploid genome.

## Background

Detailed genetic [1-3], physical mapping [4-7] and cytological studies [8,9] provided evidence for a hexaploidization event that happened in the *Brassica* lineage after divergence from the *Arabidopsis* lineage. The extant diploid *Brassica* species, such as *Brassica rapa*, *Brassica oleracea* and *Brassica nigra* are therefore characterised by a complex genome structure. *Brassica napus* (AACC) is an amphidiploid species that was formed by hybridization of two *Brassica* species that diverged approximately 4 million years ago, *Brassica rapa* (AA) and *Brassica oleracea* (CC) [10,11]. The hybridization leading to *Brassica napus* is believed to be of recent origin. Most probably it dates back less than 10,000 years. Taking the evolutionary history of the *Brassica* species into account, every single-copy gene in *Arabidopsis thaliana* should be present in three copies in the diploid *Brassica* species, in *Brassica napus* as much as six copies should be found. Microcollinearity studies revealed, however, that the triplicated genome segments that make up the diploid *Brassica* genomes have been subjected to many structural alterations, such as gene duplications, translocations, inversions, and especially frequently gene deletions. Due to these processes the gene numbers found in the *Brassica* species may show considerable deviations from the numbers stated above (summarized in [12]).

A high-quality reference sequence for the *Brassica napus* genome is not yet available, thus studies of specific regions of the *Brassica napus* genome at sequence level have relied on the analysis of bacterial artificial chromosomes (BACs) or BAC contigs. *Arabidopsis* genes were used as probes to screen a *Brassica napus* BAC library by colony hybridization for genes and/or regions of interest, subsequently the BAC clones were assigned to different loci and characterised at sequence level [13,14]. These studies showed that the A and C genomes in *Brassica napus* are very similar to those of the progenitors *Brassica oleracea* and *Brassica rapa*, however small differences with respect to the content of genes and mobile elements were observed. Thus, studies in *Brassica napus* can draw on resources that have been assembled for the progenitor genomes. For *Brassica rapa* a draft genome sequence was released [15] and for *Brassica oleracea* low coverage whole genome shotgun sequences were produced [16,17]. In addition, EST and genome survey sequences are available for different *Brassica* species (summarized in [18]).

The complex genome structure together with the high level of sequence identity of gene sequences in *Brassica oleracea* and *Brassica rapa* represent a considerable challenge for the identification of single nucleotide polymorphisms (SNPs) in *Brassica napus*[19]. Different classes of polymorphisms need to be considered. On the one hand SNPs will be found that occur between different accessions in either the A or the C genome. This class has been termed intragenomic SNP [20]. Intragenomic SNPs are readily suitable for genetic mapping and for the discrimination of accessions. Rarely SNP sites will be identified that are polymorphic in both genomes. On the other hand SNPs will be found that differentiate the homoeologous A and C genomes. This class has been referred to as interhomoeolog polymorphism [21] or intergenomic SNP [20]. Intergenomic SNPs are much more frequent than intragenomic SNPs [21], however they are neither useful for genetic mapping nor for the discrimination of accessions. Nonetheless, intergenomic SNPs may be particularly valuable for the screening of BAC libraries since they differentiate homoeologous sequences.

Initially amplicon sequencing of different accessions was used for SNP detection in *Brassica napus*[20,22,23]. Particularly large SNP collections were generated by transcriptome sequencing of different accessions [21] or by exploitation of sequence capture technology [24]. At first only few SNP markers were included in linkage maps [22,25], now high-density SNP maps are available for several *Brassica napus* mapping populations [24,26].

Illumina’s GoldenGate® Genotyping Assay exploits oligonucleotide ligation and extension assays to distinguish allelic variants [27-29]. For each SNP locus three primers are designed that bind in the vicinity of the SNP. Two allele-specific oligonucleotides which carry the discriminating bases at their 3’-ends anneal upstream of the SNP while a common locus-specific primer matches sequences located downstream of the SNP. The locus-specific oligonucleotide carries in addition an Illumicode address sequence complementary to a particular bead type. In addition to the sequences which are specific for each locus analyzed the three different primers carry at their 5’-ends sequences which are specific for a particular oligonucleotide class. After hybridization of the oligonucleotides to the DNA templates to be analyzed a polymerase fills the gap between one of the allele- and the locus-specific oligonucleotide and a ligase seals the nick so that a contiguous template is formed. These reactions are carried out for many templates and loci simultaneously. Due to the presence of sequences specific for the three different oligonucleotide classes all templates can be amplified concurrently using just three primers. The specific polymerase chain reaction (PCR) primers that correspond to the two different allele-specific oligonucleotides are labelled with Cy3 and Cy5, respectively in order to allow allele differentiation. After amplification and downstream processing the single-stranded dye-labelled products are annealed to an optical array carrying beads that are coated with sequences complementary to the Illuminocode address sequences. The genotype at a given SNP locus is determined by analyzing the ratio of Cy3 and Cy5 fluorescence. For homozygous loci in diploid organisms near pure Cy3 or Cy5 fluorescence is observed, whereas both types of fluorescence are observed for heterozygous loci. In tetraploid organisms such as *Brassica napus* the situation is more complex. Assays which are specific for one of the genomes will produce Cy3/Cy5 fluorescence ratios that are not distinguishable from those observed in diploid organisms. In contrast, assays which match target sequences on both homoeologous chromosomes may either segregate on one of the genomes or more rarely on both genomes. In these cases Cy3/Cy5 fluorescence ratios are generated that differ from those found in diploid organisms [20,30].

If BAC pools are analyzed for the presence of a particular intragenomic SNP locus with Illumina’s GoldenGate® Genotyping Assay it is expected that BAC pools that include one or more BACs corresponding to the SNP locus will show Cy3 or Cy5 fluorescence whereas BAC pools that consist solely of BACs that do not carry the locus should only give rise to residual fluorescence [31]. In case of intergenomic SNPs, BAC pools containing only one of the homoeologous loci should cause near pure Cy3 or Cy5 fluorescence whereas both types of fluorescence should be observed for those BAC pools harbouring both loci.

In this study it was tested whether Illumina’s GoldenGate® Genotyping Assay can be used for the screening of a *Brassica napus* BAC library. In particular, it was evaluated whether both inter- and intragenomic SNPs are suitable for BAC library screening.

## Results and discussion

### Isolation of *Brassica napus* gene sequences

Seven *Arabidopsis* genes (*At1g32440*, *At1g62640*, *At2g19450*, *At3g54320*, *At3g22960*, *At3g26790*, *At5g15530*) were used as probes in colony hybridization experiments to identify all corresponding sequences in a *Brassica napus* BAC library of the genotype Express. All hybridizing clones were subjected to Southern blot analysis in order to confirm the results of the colony hybridizations and to assign the BAC clones to different loci. Between two and ten *Brassica napus* loci were found for the different candidate genes, in total 32 loci were identified. For one representative clone of each locus the *Brassica napus* sequences that corresponded to the genes of interest were determined ([32]; R. Schmidt, unpublished results). All *Brassica napus* gene sequences matching a particular *Arabidopsis* candidate gene were aligned and based on these alignments gene-specific oligonucleotide pairs were developed.

### Multidimensional screening platform of the *Brassica napus* BAC library

**Figure 1:**
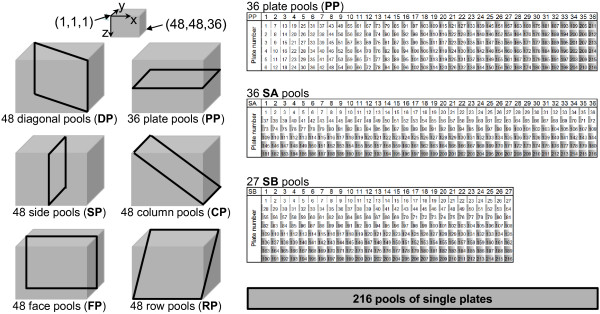
**Multidimensional BAC pooling.** Two-hundred sixteen 384-well microtiter plates were arranged in a cube consisting of 36 layers of six plates each. The clones were pooled according to their position in the cube along the six distinct coordinate axes. In total, 276 six-dimensional pools were generated. The SA and SB pools were generated according to the scheme shown by combining clones of six and eight plates, respectively. For the screening of all eight dimensions 339 pools had to be analyzed. An additional dimension consisted of 216 pools that contained clones of single 384-well microtiter plates.

The 82,944 clones of a *Brassica napus* BAC library were pooled according to the scheme shown in Figure 1. Using this pooling system every clone of the library was represented once in each of six dimensions [33]. The 24 *Brassica napus* loci analyzed in this work were represented on average 10.6 times in the BAC library. Unambiguous deconvolution of clone coordinates using the six-dimensional pooling system depends on the depth of representation of a particular locus in the BAC library. In order to facilitate BAC coordinate deconvolution additional pooling dimensions were established. The SA and SB pools contain clones of six and eight 384-well plates, respectively (Figure 1). Moreover, pools containing clones of single plates with 384 BAC clones each were established. For library screening the six-dimensional pools were either analyzed together with the SA and SB pools (eight screening dimensions) or with the 216 single plate pools (seven screening dimensions).

In order to check the suitability of the established BAC pools for PCR-based screening, three gene-specific oligonucleotide pairs were used for PCR analyses with DNA samples of all six-dimensional BAC pools as well as the SA and SB pools as templates. Based on the results of the colony hybridization and locus assignment studies the pools which should harbour a particular gene-specific amplicon were deduced and served as reference for the results obtained by PCR. The predictions were found to be in good agreement with the experimental results (data not shown). Hence, both size and DNA quality of the different pools were suitable for PCR-based screening.

### Screening of the *Brassica napus* BAC library with Illumina's GoldenGate® genotyping assay

In the majority of cases it was attempted to analyze the BAC pools for the presence/absence of individual *Brassica napus* genes. For each intragenomic SNP assay a single gene was assessed whereas intergenomic assays should allow the simultaneous detection and differentiation of homoeologous genes. The data set used in this study consisted of twenty intergenomic and fourteen intragenomic assays. In five additional cases the assays were designed in such a way that homoeologous genes would not be discriminated (Additional file 1, Additional file 2).

The 31 assays included in oligonucleotide pool assay 1 (OPA1) (Additional file 1, assays without the name affix “A”) were used to screen all six-dimensional as well as the SA and SB BAC pools. DNA samples of *Brassica* accessions or pools of accessions were also analyzed.

**Figure 2:**
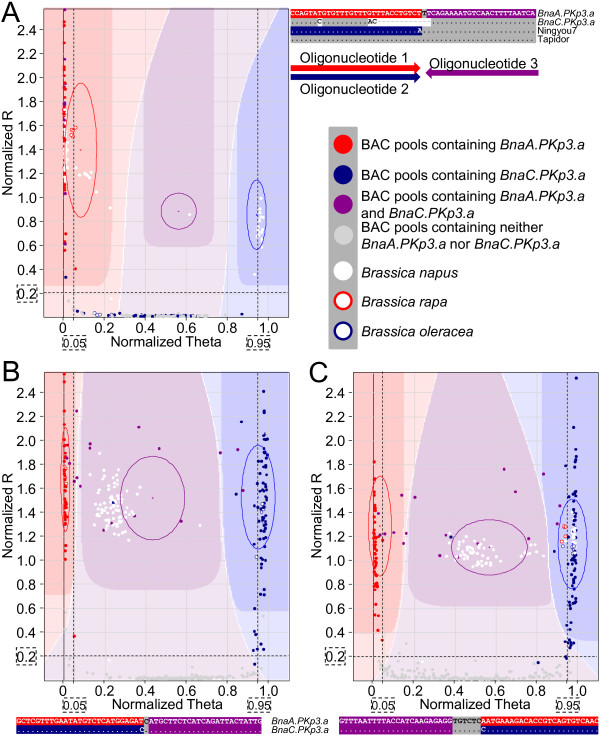
**Analysis of BAC pools and *Brassica *accessions with oligonucleotide pool assays.** The sequences represent those that were established for the genes found in the Express genome unless indicated otherwise. Panel **(A)** shows the results for SNP assay #52 which was specific for *BnaA*.*PKp3*.*a*. In this intragenomic SNP assay oligonucleotide 1 corresponds to an allele which was found for example in the genotypes Express and Tapidor, whereas oligonucleotide 2 matched the allele present in the Ningyou7 genome. The darker colored regions in the graphs correspond to genotype call areas which were defined by GenomeStudio Data Analysis Software v2011.1, in the red and blue areas all plants homozygous for the Express and Ningyou7 alleles were found, respectively. Heterozygous plants showed up in the purple area. The results for SNP assays #51 and #47 are displayed in panels **(B)** and **(C)**, respectively. Oligonucleotides 1 and 2 of intergenomic assays #51 and #47 discriminated between homoeologous genes *BnaA*.*PKp3*.*a* and *BnaC*.*PKp3*.*a*. In all graphs the data points for the BAC pools were color-coded according to the information which was deduced from the BAC coordinates that had been identified via colony hybridization and locus assignment studies. The dashed lines and boxed values correspond to the thresholds which were implemented for the optimized scoring method.

SNP calling was performed with the Genotyping Module in GenomeStudio Data Analysis Software v2011.1. After normalization, signal intensities (normalized R) and allele frequencies (normalized Theta) were plotted for all samples and assays. For each assay, matrices of signal thresholds were defined for the different genotypes using a clustering algorithm also referred to as genotype call areas. Based on this information the SNPs were called for the different samples. Examples are shown in Figure 2.

Luo et al. [31] reported that reliable scoring of BAC pools with oligonucleotide pool assays was possible provided that genotype call areas obtained for an *Aegilops tauschii* F_2_ population served as reference for the SNP calling of *Aegilops tauschii* BAC pools. This strategy was adapted for the data analysis in this study. In order to allow analysis of intra- and intergenomic SNPs DNAs of *Brassica* accessions rather than DNAs of a mapping population of *Brassica napus* were included as reference samples. In total, DNAs of forty different *Brassica napus* accessions, one *Brassica napus* F_1_-plant and four pools of *Brassica* accessions were analyzed. Genotype call areas were initially established based on the results of 34 *Brassica* DNA samples by the GenomeStudio Data Analysis Software v2011.1. These were then exported and used for the analysis of all samples. If appropriate, genotype call areas were manually adjusted.

**Figure 3:**
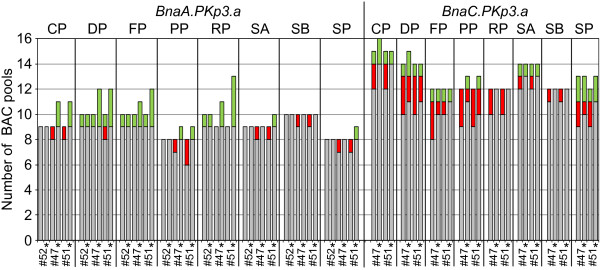
**Scoring of BAC pools for the presence of SNPs with two methods.** The coordinates of BAC clones which were shown to contain genes *BnaA*.*PKp3*.*a* and *BnaC*.*PKp3*.*a* according to the results of colony hybridizations and locus assignment studies served as reference for the scoring of the BAC pools with oligonucleotide pool SNP assays. The results of the optimized scoring method (for details please refer to the text) are marked with stars the other data were obtained with the clustering algorithm in GenomeStudio Data Analysis Software v2011.1. Grey shading indicates the number of BAC pools that were identified with a particular SNP assay (#…) and corresponded to known BAC coordinates. Red boxes refer to the number of BAC pools representing known BAC coordinates but that were not detected with the SNP assays. Green shading highlights the number of identified pools that did not coincide with known BAC coordinates. The pools of the different dimensions are abbreviated in the same way as shown in Figure 1.

The data for SNP assays #52, #51, #47 that were developed for the analysis of two homoeologous genes, *BnaA.PKp3.a* and *BnaC.PKp3.a*, are shown as examples (Figure 2). The results of gene-specific assay #52 (Figure 2A) were found to be in good agreement with the predictions based on the known coordinates that had been previously identified using colony hybridizations since all 71 pools which were known to contain *BnaA.PKp3.a* were determined (Figure 3). In addition, three pools which do not match any known BAC coordinate were also identified and classified as false positive scores. The genotyping results obtained for the pools of *Brassica rapa* and *Brassica oleracea* accessions were used to assign the different *Brassica napus* genes to the progenitor genomes (Additional file 1). Figure 2B shows that the SNP of assay #51 which is specific for gene *BnaA.PKp3.a* is present in the *Brassica rapa* accessions tested, whereas *Brassica oleracea* genotypes match the SNP specific for *BnaC.PKp3.a*. For gene-specific assay #52 the *Brassica oleracea* accessions only give rise to residual fluorescence whereas the *Brassica rapa* accessions harbour the SNP specific for gene *BnaA.PKp3.a*.

The clustering algorithm did not perform equally well in case of intergenomic SNPs. The results for assays #51 and #47 exemplify this (Figure 2B and 2C). Assays #47 and #51 correctly determined 93% and 90%, respectively of BAC pools that contained locus *BnaA.PKp3.a*, for locus *BnaC.PKp3.a* lower detection rates of 82 and 85%, respectively resulted (Figure 3). The eighteen BAC pools in which both homoeologous loci were present were in several cases not correctly assigned using the clustering algorithm, instead such pools were often ascribed to the call areas representing a single locus only (Figure 2B and 2C).

In contrast, the clustering algorithm was well-suited to identify plants that carried both loci of a particular intergenomic SNP. *Brassica napus* accessions were expected to contain both homoeologs, and indeed the clustering algorithm correctly assigned all *Brassica napus* accessions to the call area which corresponded to both SNPs in case of assay #51 (Figure 2B). Also in case of intergenomic assay #47 most *Brassica napus* accessions were found in the call area which indicated the presence of both SNPs in these plants (Figure 2C). However, some *Brassica napus* accessions were found to be homozygous for the SNP specific for *BnaC.PKp3.a.* These results were consistent with the finding that these *Brassica napus* accessions harboured an allele of *BnaA.PKp3.a* that was identical in sequence to *BnaC.PKp3.a* in the gene region corresponding to oligonucleotides 2 and 3 of assay #47 (R. Schmidt, unpublished results). With this particular assay the pools of *Brassica rapa* and *Brassica oleracea* accessions were assigned to the same genotype. Moreover, the sequences of oligonucleotides 2 and 3 of assay #47 perfectly match to the WGS sequences of *Brassica rapa* Chiifu-401 and *Brassica oleracea* O212 [34]. These results illustrate that intergenomic SNPs that were identified based on A and C genome sequences of few *Brassica napus* accessions are not necessarily capable to distinguish generally between A and C genomes.

The results for the plant samples presented here are consistent with other more comprehensive studies which showed that genotype calling of plant samples by the GenomeStudio clustering algorithm is reliable and efficient (*e.g.*[20,31]), hence this methodology is widely used. In contrast, Luo et al. [31] concluded that the scoring of BAC pools was much more error-prone unless call areas established for a population of F_2_ plants provided a reference for the genotyping of BAC pools. In our study a considerable fraction of BAC pools were not assigned to the correct call areas with intergenomic SNPs, despite the fact that 45 *Brassica* DNA samples were included in the genotyping experiments. The data presented in Figure 2B and 2C show that an adjustment of genotype call areas which would correctly assign all BAC pools to the different genotype classes is not possible because some of the normalized Theta values that belong to BAC pools that should give a signal with both SNPs are intermingled with those of BAC pools that harbour only one of the analyzed loci.

Loci and/or alleles are present in the vast majority of cases in certain ratios in a given plant accession whereas individual BAC clones will be present in unequal ratios in any given BAC pool. Furthermore, individual pools may not harbour equal numbers of BACs containing the homoeologous loci. These differences may account for the obvious discrepancy that has been detected with respect to scoring reliability of BAC pools and plant samples.

### Optimized SNP scoring method for BAC pools

In order to improve the SNP genotyping of BAC pools an alternative scoring method was devised and evaluated. Only BAC pools which gave rise to normalized fluorescence intensity equal to or above 0.2 were classified as containing an SNP locus. Signals with normalized Theta values in the range from 0.05 to 0.95 were assigned to the category containing both SNPs, whereas values equal to or below 0.05 and equal to or above 0.95 were categorised to contain the alternative SNPs (Figure 2). These thresholds for the normalized Theta values were chosen based on the data that were obtained for 14 intragenomic assays (Additional file 2B) and for five assays that did not discriminate between homoeologous genes (Additional file 2C). The majority of BAC pools which showed fluorescence intensities equal to or above 0.2 had normalized Theta values between 0 and 0.05 or 0.95 and 1.0, only 6% of the values were found in the range from 0.05 and 0.95 (Additional file 3A). An analysis of the individual assays revealed, however, differences. For seven assays none of the values were found in the interval between 0.05 and 0.95, for another seven assays less than 2.5%, but in two out of the 19 assays approximately 30% of values were observed in this interval (Additional file 3B).

Figures 2B, 2C and 3 show that several clone pools which were known to contain the loci of interest were identified with the optimized scoring system but not with the clustering algorithm. With the clustering algorithm 12 and 14% of the expected BAC pools were not correctly assigned to the genotype call areas with assays #51 and #47, respectively. With the alternative scoring only 3% of the BAC pools were misclassified with each of the two assays.

The identification of a BAC coordinate requires a score in each of the screening dimensions, a scoring failure or a wrong SNP assignment in any of the dimensions will result in the complete loss of this particular BAC coordinate from the list of putative coordinates. Twenty-five BACs were known to carry loci *BnaA.PKp.a* or *BnaC.PKp.a* (Table 1). Using the clustering algorithm assays #51 and #47 identified only 11 (44%) and 14 (56%) BAC coordinates, respectively whereas the optimized scoring method revealed for assay #51 22 (88%) and for assay #47 23 (92%) coordinates. Thus, with the proposed alternative scoring method the frequency of false negative BAC coordinates was very much reduced. Moreover, this scoring scheme can be used independently of plant reference samples. Taking advantage of this, for the analysis of OPA2 a much smaller set of plant DNA samples was evaluated. In these experiments only the *Brassica napus* accession Express, the four different pools of *Brassica* accessions and two *Brassica oleracea* and *Brassica rapa* accessions each were examined.

**Table 1:** **Summary of SNP screening results for the *Brassica napus *BAC library**

**Gene(s)**	**Number of known BACs**	**Number of confirmed BACs (% of known BACs)**	**Number of putative BACs**	**Number of confirmed BACs (% of putative BACs)**	**Assay**	**Gene(s)**	**Number of known BACs**	**Number of confirmed BACs (% of known BACs)**	**Number of putative BACs**	**Number of confirmed BACs (% of putative BACs)**
*BnaA.PKp3.a*	10	10 (100%)	14	10 (71.4%)	#52					
*BnaA.PKp3.a*	10	10 (100%)	19	10 (52.6%)	#51	*BnaC.PKp3.a*	15	12 (80%)	38	12 (31.6%)
*BnaA.PKp3.a*	10	10 (100%)	17	10 (58.8%)	#47	*BnaC.PKp3.a*	15	13 (86.7%)	35	13 (37.1%)
*BnaA.KAS III.a*	0	0	0	0	#7	*BnaC.KAS III.a*	4	4 (100%)	5	4 (80%)
*BnaA.KAS III.a*	0	0	0	0	#9	*BnaC.KAS III.a*	4	4 (100%)	5	4 (80%)
					#2	*BnaC.DGAT1.a*	15	13 (86.7%)	39	13 (33.3%)
*BnaA.DGAT1.a*	2	1 (50%)	1	1 (100%)	#4	*BnaC.DGAT1.a*	16	14 (87.5%)	30	14 (46.7%)
*BnaA.DGAT1.a*	2	1 (50%)	1	1 (100%)	#5	*BnaC.DGAT1.a*	16	15 (93.8%)	33	15 (45.5%)
*BnaA.DGAT1.b*	16	13 (81.3%)	33	13 (39.4%)	#9A	*BnaC.DGAT1.b*	1	0	0	0
*BnaA.DGAT1.b*	16	13 (81.3%)	38	13 (34.2%)	#10A	*BnaC.DGAT1.b*	1	1 (100%)	1	1 (100%)
					#13A	*BnaC.DGAT1.c*	10	9 (90%)	10	9 (90%)
					#14A	*BnaC.DGAT1.c*	10	7 (70%)	7	7 (100%)
*BnaA.WRI1.a*	5	4 (80%)	5	4 (80%)	#32	*BnaC.WRI1.a*	9	8 (88.9%)	11	8 (72.7%)
*BnaA.WRI1.a*	5	4 (80%)	6	4 (66.7%)	#37	*BnaC.WRI1.a*	9	8 (88.9%)	11	8 (72.7%)
*BnaA.WRI1.b*	14	13 (92.9%)	36	13 (36.1)	#28	*BnaC.WRI1.b*	11	11 (100%)	52	11 (21.2%)
*BnaA.WRI1.b*	14	14 (100%)	534	14 (2.6%)	#30	*BnaC.WRI1.b*	11	2 (18.2%)	13	2 (15.4%)
*BnaA.PKp1.a*	9	0	0	0	#7A					
*BnaA.PKp1.a*	9	1 (11.1%)	2	1 (50%)	#57	*BnaC.PKp1.a*	7	7 (100%)	10	7 (70%)
*BnaA.PKp1.a*	9	0	0	0	#60	*BnaC.PKp1.a*	7	5 (71.4%)	16	5 (31.3%)
*BnaA.PKp1.a*	9	2 (22.2%)	2	2 (100%)	#3A	*BnaC.PKp1.a*	7	4 (57.1%)	4	4 (100%)
*BnaA.PKp1.a*	9	3 (33.3%)	3	3 (100%)	#8A	*BnaC.PKp1.a*	7	2 (28.6%)	2	2 (100%)
					#58	*BnaC.PKp1.a*	7	3 (42.9%)	3	3 (100%)
					#55	*BnaC.PKp1.b*	18	11 (61.1%)	45	11 (24.4%)
					#56	*BnaC.PKp1.b*	18	13 (72.2%)	50	13 (26%)
*BnaA.FUS3.a*	18	17 (94.4%)	108	17 (15.7%)	#14	*BnaC.FUS3.a*	20	13 (65%)	77	13 (16.9%)
*BnaA/C.FUS3.a*	38	36 (94.7%)	2492	36 (1.4%)	#10					
*BnaA/C.FUS3.a*	38	37 (97.4%)	3047	37 (1.2%)	#12					
*BnaA/C.FUS3.a*	38	36 (94.7%)	2249	36 (1.6%)	#18					
*BnaA/C.FUS3.a/b*	62	58 (93.5%)	12114	58 (0.5%)	#25					
*BnaA.FUS3.b*	8	5 (62.5%)	10	5 (50%)	#20	*BnaC.FUS3.b*	16	16 (100%)	38	16 (42.1%)
*BnaA.FUS3.b*	8	8 (100%)	12	8 (66.7%)	#21	*BnaC.FUS3.b*	16	14 (87.5%)	33	14 (42.4%)
*BnaA.FUS3.b*	8	5 (62.5%)	9	5 (55.6%)	#22	*BnaC.FUS3.b*	16	16 (100%)	67	16 (23.9%)
*BnaA.BCCP2.a*	12	11 (91.7%)	19	11 (57.9%)	#45					
*BnaA.BCCP2.a*	12	10 (83.3%)	17	10 (58.9%)	#70					
					#43	*BnaC.BCCP2.a*	13	10 (76.9%)	36	10 (27.8%)
					#44	*BnaC.BCCP2.a*	13	11 (84.6%)	39	11 (28.2%)
*BnaA.BCCP2.b*	9	9 (100%)	10	9 (90%)	#2A					
*BnaA/C.BCCP2.b*	21	20 (95.2%)	337	20 (5.9%)	#65					
					#41	*BnaC.BCCP2.b*	12	11 (91.7%)	99	11 (11.1%)

In one of the BAC clones which contained locus *BnaC.DGAT1.a* only a segment of the gene was present, therefore identification of this clone was only possible with assays #4 and #5 but not with #2.

**Figure 4:**
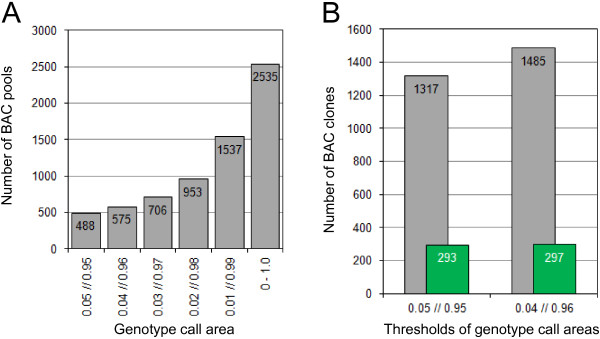
**Defining the thresholds of the genotype call areas.** The bars in panel **(A)** show how the thresholds for the genotype call areas affected the number of BAC pools that were assigned to the category containing both SNPs. In panel **(B)** the grey bars show the total number of putative BAC clones which resulted if two different sets of thresholds were applied to define the genotype call areas. The green bars correspond to the BAC clones which were confirmed to contain the loci of interest. Results for the 20 intergenomic assays listed in Additional file 2A were taken into account for the analysis represented in the two panels.

The thresholds that were chosen to delineate the area which was categorised to carry both SNPs constituted a compromise. Figures 2 and 3 reveal that even the optimized scoring system did not identify all pools that harbour both loci. This could be fixed if the area which was classified to contain both SNPs would be increased. The consequences of an increased scoring area are seen in Figure 4. All 20 intergenomic SNPs that were analyzed in this study (Additional file 2A) were evaluated and it was calculated how many BAC pools would be classified as carrying both loci. Changing the scoring range from 0.05-0.95 to 0.04-0.96 resulted in 87 additional pools which were classified as containing both loci (Figure 4A). Due to this the number of putative BAC clones which needed to be assessed for the presence of the loci in additional experiments was increased by approximately 13%. Thus, considerable more screening effort was needed whereas the number of clones that could be confirmed changed only marginally it increased by less than 1.5% (Figure 4B). Based on these results the thresholds for the genotype call areas were placed for all subsequent analyses at 0.05 and 0.95.

### Assessment of screening results

The putative BAC clones that were discovered with the oligonucleotide pool assays were analyzed with appropriate gene-specific amplicons in order to identify those clones that carried the loci of interest. A comparison of the coordinates of clones which were confirmed to contain the assayed loci with the list of BAC clones known to carry the loci of interest (Table 1, Additional file 2) revealed that multiplex PCR screening recovered 81.6% of the known BAC coordinates. The screening success of intergenomic SNPs was with 77.1% in the same range as that of gene-specific SNPs (76.2%). The five assays which did not discriminate between homoeologous genes in the Express genome even recovered 94.9% of the known clones.

For the experiments with OPA1 all six-dimensional pools as well as the SA and SB pools were screened, for the experiments with OPA2 all six-dimensional pools and the 216 single-plate pools were assessed. Thus, seven and eight screening dimensions were analyzed with OPA2 and OPA1, respectively. Taking into account that the identification of BAC coordinates relied on a score in each of the dimensions analyzed an overall success rate of more than 80% has to be regarded as very high. It can be concluded that a reliable screening platform has been established.

**Figure 5:**
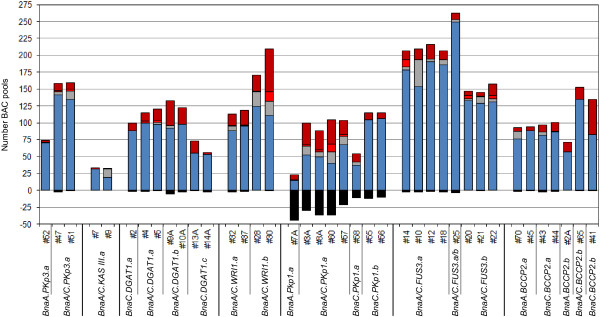
**Summary of screening results for BAC pools of the *Brassica napus *BAC library.** The black boxes represent the pools of known BAC coordinates which were not scored because their normalized R value was lower than 0.2. The total area covered by the blue, grey, red and brown bars corresponds to the total number of BAC pools for which genotypes were assigned with the different SNP assays (#…) used. BAC pools that belonged to known BAC coordinates are shown as blue, grey and red bars. Blue bars correspond to pools for which the SNP assignment was consistent with that expected for the known coordinates. The grey bars represent pools which were assigned to the class containing both SNPs, whereas a single SNP would be predicted based on the information for the known clones. Pools with a SNP classification that caused the omission of known BAC coordinates from the list of putative clones are shown as red bars. The brown bars represent pools that did not belong to known BAC coordinates.

On average, only 5.8% of the pools which corresponded to known BAC coordinates failed to be detected because their normalized R values did not surpass the applied threshold. The summary of the screening results (Table 1) shows that not all loci were detected equally well. For example, the screening success for the *BnaX.PKp1*-loci, in particular for *BnaA.PKp1.a*, was lower than for the other genes of interest. For the assays corresponding to the *BnaX.PKp1*-loci the fraction of pools that escaped detection ranged from 9% for assay #56 to 75% for assay #7A (Figure 5). As a consequence two of the assays (#60, #7A) designed for *BnaA.PKp1.a* did not reveal any of the nine BAC clones that contained this locus, three other assays (#57, #3A, #8A) revealed between one and three of the known BAC coordinates (Table 1, Additional files 2A and 2B). When it was attempted to recover the BAC clones containing *BnaA.PKp1.a* from the original glycerol stocks only few colonies could be retrieved whereas for most other clones many colonies were recovered when the glycerol stocks were spread on agar plates. These results suggest that clones that do not grow vigorously may be underrepresented in the pools and hence may escape detection.

In approximately half of the cases in which BAC clones were not identified, only one of the screening dimensions failed. In some instances false negative scores for a certain pool were observed with two or more independent assays corresponding to a particular gene whereas assays developed for other genes faithfully detected this pool. The BAC pools were established manually therefore it is possible that in rare cases individual clones were erroneously omitted from a pool and/or allocated to a wrong pool.

Assays #2, #4 and #5 were designed to detect *BnaC.DGAT1.a* (Table 1, Additional files 1, 2A-B). For fifteen out of the sixteen clones known to contain this gene positive signals were found in at least seven out of the eight screening dimensions with all three assays. In contrast, clone 202N5 was only detected with assays #4 and #5, whereas assay #2 did not reveal any signal specific for this clone. Analyses with gene-specific PCR amplicons confirmed that clone 202N5 carried sequences corresponding to assays #4 and #5. In contrast, sequences downstream of this region and where assay sequences #2 were located were not present. This example shows the potential of multiplex-PCR screening for BAC contig building.

### Evaluation of the optimized SNP scoring method

The reliability of the optimized scoring system was evaluated based on the entire data set (Figure 5). On average 78% of all pools were assigned to the genotype that was expected for the known BAC coordinates. Another 7% of the pools were assigned to both SNPs although the known BAC coordinates would predict only one of the SNPs. This type of SNP assignment did not lead to the omission of a known BAC coordinate from the list of putative clones, but in case of intergenomic SNPs it may lead to an increase in the number of putative clones that are identified. Cases in which the scoring method assigned a SNP that was in conflict with the expected genotype were rare, only 2% of the genotype scores were affected. Approximately 13% of the pools that were assigned to a genotype did not correspond to known BAC coordinates this type of false positive scores may increase the number of putative clones.

### Considerations for SNP assay design

In this study all gene sequences corresponding to a particular *Arabidopsis thaliana* candidate gene were taken into consideration to select gene regions most suitable for SNP assays. The assays were developed such that the gene(s) and or homoeologs to be tested showed several mismatches and/or indels as close to the assay site as possible when compared to other homologs present in the *Brassica napus* genome. Consequently, overall an acceptable rate of false positive scores was observed, only few assays showed a high frequency of false positive scores. For assays #41 and #30 the proportion of false positive scores was particularly high, it accounted for approximately 40% of the detected pools (Figure 5).

With assay #30 534 and 13 putative coordinates were detected for *BnaA.WRI1.b* and *BnaC.WRI1.b*, respectively. In contrast, assay #28 revealed for the same loci 36 and 52 putative coordinates. With assay #28 all 11 BAC clones known to be present for locus *BnaC.WRI1.b* in the library were detected, whereas assay #30 revealed only 2 BAC clones for this locus (Table 1). Detailed analysis of the screening data for assay #30 showed that 14 pools that should give a signal for *BnaC.WRI1.b* were only assigned to the category specific for *BnaA.WRI1.b.* These results suggest that the combination of oligonucleotides 1 and 3 of assay #30 did not only detect BAC clones containing *BnaA.WRI1.b* but also additional loci that gave rise to scores that did not match known BAC coordinates of *BnaA.WRI1.b* and interfered with the correct assignment of pools containing *BnaC.WRI1.b*. Among the list of putative BAC clones which were revealed by assay #30 for *BnaA.WRI1.b* several BAC coordinates were found that carried loci *BnaA.WRI1.a* and *BnaC.WRI1.a*. This implies that oligonucleotides 1 and 3 identified BAC pools containing *BnaA.WRI1.a* and *BnaC.WRI1.a*, although the assay sequences do not perfectly match to the sequences of these two loci. For oligonucleotide 3 one indel and one SNP were observed, both of which were more than 10 bp upstream of the assay nucleotide. In case of oligonucleotide 1 an indel and a mismatch were found 6 and 9 bp downstream of the assay nucleotide. The detailed analysis of the results for assay #30 stress that assay sequences should preferably contain multiple mismatches and/or indels very close to the assay site when compared to other homologs in order to avoid that homologous sequences interfere with the assay sequences in BAC library screens.

For many of the loci of interest the screen of the multidimensional screening platform resulted in a manageable list of putative BAC coordinates (Table 1). Thus, the use of seven or eight screening dimensions as described for OPA2 and OPA1 is suitable for a library with estimated 10-fold genome coverage. For loci for which ten or fewer BAC coordinates were known to be present in the library, in all but one case (#60 for locus *BnaC.PKp1.a*) the fraction of clones that could be confirmed to contain the loci of interest made up 50% or more of the putative coordinates. As expected, this fraction decreased if loci with more known coordinates were evaluated. The list of putative coordinates ranged from 13 to 108 when the loci were present between 11 and 20 times in the library, with the notable exception of data that were obtained with assay #30 for locus *BnaA.WRI1*.*b* and that were already discussed. Assays that did not discriminate between homoeologs and that were aimed to detect between 21 and 62 coordinates revealed many hundred (#65) or several thousand putative BAC coordinates (#10, #12, #18, #25). These results emphasize that it is essential to discriminate between homoeologous genes if a BAC library of a complexity similar to the one described here is used for PCR-based analyses.

## Conclusions

Screening with multiplex SNP assays was successfully applied in the polyploid species *Brassica napus* for a BAC library with estimated 10-fold genome coverage. Intra- and intergenomic SNPs were equally suited for this purpose, provided that an optimized SNP calling method was implemented. Owing to the 10-fold coverage of the library only assays differentiating between (paleo)homoeologous genes proved to be suitable. For assays not discriminating between (paleo)homoeologous genes, even the use of one or two extra screening dimensions in addition to six-dimensional pools did not result in manageable lists of putative BAC coordinates. Our results suggest that BAC library screening with multiplex SNP assays can be effectively applied in polyploid species if sufficient sequence information is available for the identification of suitable SNPs.

## Methods

### Isolation and characterisation of *Brassica napus* gene sequences corresponding to selected *Arabidopsisthaliana* genes

Standard molecular biology techniques were performed as described in Sambrook et al. [35].

A *Brassica napus* BAC library of the genotype Express was constructed at Keygene N.V. (Wageningen, The Netherlands). The library consisted of almost 83,000 clones, arranged in 216 384-well microtiter plates. All clones of the library were gridded in duplicate and at high density on nylon membranes. Colony hybridizations using PCR-amplified Arabidopsis genes as probes were carried out according to O’Neill and Bancroft [4]. The DNA of all hybridizing clones was isolated, restricted with appropriate enzymes and subjected to Southern blot analyses with the *Arabidopsis thaliana* gene sequences as probes. Based on the resulting patterns the different BAC clones were assigned to different loci.

The analysis of 24 loci revealed an approximate 10-fold genome coverage for the BAC library. For *Brassica napus* genome size estimates of 1130 to 1235 Mbp were reported [36]. Consequently, the inserts of the BAC library should span on average between 135 and 150 kbp.

A representative BAC clone for each locus was selected for subcloning and sequencing of the *Brassica napus* genes that corresponded to a particular *Arabidopsis thaliana* candidate gene. Sequencing was performed by primer walking and carried out at Eurofins MWG Operon (Ebersberg, Germany) or at the IPK Gatersleben (Germany). Oligonucleotides were purchased from Eurofins MWG Operon (Ebersberg, Germany). Nomenclature of the genes followed the standard proposed by Østergaard and King [37].

### Development of gene-specific amplicons

All *Brassica napus* gene sequences that corresponded to a particular *Arabidopsis thaliana* candidate gene were aligned using the CHAOS + Dialign webserver [38]. These alignments provided the basis for the design of gene-specific oligonucleotide pairs. Primers with an optimum melting temperature of 60°C were selected with Primer3 (Whitehead Institute for Biomedical Research, Cambridge MA; [39]).

DNA samples of BAC clones that represented all different *Brassica napus* loci corresponding to a particular candidate gene were used as templates in PCR experiments in order to identify the annealing temperature at which gene-specific products were obtained for each oligonucleotide pair. These optimized temperature profiles were then used for PCR amplification with total DNA of the cultivar Express as template. Only if direct sequencing of the resulting PCR product confirmed that a particular primer pair amplified a fragment of a single gene it was included in the genetic diversity studies.

### SNP discovery

DNA samples derived from a set of *Brassica napus* accessions served as templates for PCR amplifications with the gene-specific oligonucleotide pairs and the PCR products were directly sequenced. The resulting sequences were manually edited and subsequently aligned with BioEdit [40]. The sequence alignments were inspected for the presence of SNPs. The genetic diversity studies were performed for homoeologous *Brassica napus* genes, it was therefore possible to identify and distinguish intragenomic and intergenomic SNPs. The results of the genetic diversity studies will be reported elsewhere.

### Multidimensional pooling strategy

A six-dimensional pooling strategy as described by Klein et al. [33] was adopted for the *Brassica napus* BAC library of the genotype Express. The 216 384-well microtiter plates that make up the BAC library were conceptually arranged in a cube consisting of 36 layers, 48 columns, and 48 rows, thus each layer consisted of six 384-well plates. The clones were then pooled according to their position in the cube along the six distinct coordinate axes to generate 36 plate pools (PP), 48 diagonal pools (DP), 48 side pools (SP), 48 column pools (CP), 48 face pools (FP) and 48 row pools (RP). The plate pools consisted of 2304 clones each, whereas 1728 clones each made up the pools of the other five dimensions (Figure 1).

Plate pools were generated by transferring clones from the six 384-well plates which make up one layer of the cube with a 384-pin tool into a single plate. To generate the pools of the other dimensions in the most efficient manner first suitable master pools were assembled which contained all clones of up to six particular microtiter plates. For the construction of the master plates for the side pools, face pools and diagonal pools, for example, all plates which were present in the same position in six consecutive layers of the cube were combined. Depending on which of the dimensions was prepared 24-, 16-well pin tools or toothpicks were used to transfer the appropriate BAC clones from the master pool plates into the stock plate for each particular pool.

Stocks for each individual pool were generated in a single 384-well microtiter plate containing 70 μl of glycerol-containing bacterial growth medium plus 12.5 μg/ml chloramphenicol per well. After incubation for 16–20 h at 37°C all cultures of a single 384-well microtiter plate were combined in sterile containers and aliquots were stored at −80°C until further use.

For the SA and SB pools clones of six (36 pools à 2304 BAC clones) and eight entire microtitre plates each (27 pools à 3072 BAC clones) were combined according to the scheme shown in Figure 1. In addition, 216 pools containing clones of single plates with 384 BAC clones each were established.

### BAC pool DNA isolation

The glycerol stocks of the individual pools were used to inoculate 100 ml of liquid bacterial growth medium supplemented with 12.5 μg/ml chloramphenicol. Growth took place for 16–20 h in a gyratory shaker at 37°C. BAC DNA isolation was performed with NucleoBond**®** PC100 (Macherey-Nagel GmbH & Co. KG, Düren, Germany) according to manufacturer’s instructions for the six-dimensional pools as well as the SA and SB pools. For the 216 DNA pools that were generated from single plates 5 ml of bacterial culture each were used for DNA extractions with the GeneJET™ Plasmid Miniprep Kit (Fermentas, St. Leon Rot, Germany). Quantification of the DNA samples was performed with the Quant-iT™ PicoGreen® dsDNA Assay Kit (Life Technologies GmbH, Darmstadt, Germany).

### PCR conditions

PCR was carried out in reaction volumes of 30 μl containing approximately 5 ng BAC-pool DNA, 1 × DreamTaq™ buffer, 250 μM dNTP, 30 pmol of each primer and 1 U of DreamTaq**®** DNA Polymerase (Fermentas, St. Leon Rot, Germany). All samples were preheated for 10 min at 95°C and then subjected to 35 PCR cycles. The cycles consisted of a denaturation step at 94°C for 30 s, an annealing step for 30 s, and an elongation step at 72°C for 1 min. After a final extension of 5 min at 72°C the reactions were cooled down to 15°C. Aliquots of 5 μl were resolved on agarose gels in 1 × TBE.

In order to assay single BAC clones for the presence or absence of a particular amplicon single bacterial colonies were transferred to PCR tubes containing all necessary reagents. Conditions for PCR were the same as described above.

In case *Brassica napus* DNA samples were used for PCR the reactions were carried out in 50 μl and approximately 20 ng of template DNA were used for amplification. Prior to sequencing the PCR products were purified with appropriate spin columns (peqGOLD MicroSpin Cycle-Pure Kit, PEQLAB Biotechnologic GmbH, Erlangen, Germany).

### Analysis with Illumina’s GoldenGate® genotyping assay

Sequences containing suitable SNPs were extracted and supplied to Illumina Inc. (San Diego, CA) for design of multiplex oligonucleotide pool assays (OPA, Additional file 1). The 31 assays without the name affix “A” were part of OPA1, the remaining eight assays were part of OPA2. All assays used had a designability score higher than 0.6.

All experimental steps were carried out as recommended by the manufacturer and the fluorescence signals were recorded with a BeadXpress Reader (Illumina Inc. San Diego, CA).

Initially three different DNA template amounts were tested for 20 BAC pools each in a multiplex GoldenGate® Genotyping Assay. Similar results were obtained regardless whether 50, 125 or 250 ng were used as DNA input. In all subsequent experiments 125 ng template DNA were used for each pool that contained between 1728 and 3072 clones. For all BAC pools that harboured DNA of 384 clones each lower amounts of template DNA were used. Tests with six such BAC pools showed that comparable results were obtained with 5, 10 and 25 ng of template DNA. In all subsequent experiments 10 ng of template DNA served as DNA input for the BAC pools of single 384-well plates.

Plant DNA samples were amplified with the Illustra Genomi Phi V2 DNA Amplification Kit (GE Healthcare Europe GmbH, Munich, Germany) and 250 ng each of the resulting reaction products were taken as templates for genotyping**.**

For the experiments with OPA1 all six-dimensional pools as well as the SA and SB pools were analyzed alongside with DNA of 40 *Brassica napus* accessions, a *Brassica napus* F_1_-plant and four pools of *Brassica* accessions. Two of the pools consisted of summer and winter oilseed rape lines, the other two pools were comprised of *Brassica oleracea* and *Brassica rapa* accessions. Initially, the GenomeStudio Data Analysis Software v2011.1 (Illumina Inc., San Diego, CA) was used for calling of genotypes with default parameters. The genotype call areas were initially defined based on the analysis of 29 DNAs of *Brassica napus* accessions, one F_1_-plant and the four different DNA pools of *Brassica* accessions. The clusters for the three different genotypes were exported from the plots to serve as reference for the analysis of the BAC pool DNA samples. Based on the data of all DNA samples the genotype call areas were manually adjusted, if needed.

Subsequently, an optimized scoring system for BAC pools was developed. Only BAC pools which showed normalized fluorescence intensities (Normalized R) equal to or higher than 0.2 were classified as carrying a particular SNP. BAC pools showing signals with normalized Theta values (Normalized Theta) equal to or below 0.05 and equal to or above 0.95 were classified to contain the alternative SNPs, whereas values in the range from 0.05 to 0.95 were assigned to the category containing both SNPs.

For the experiments with OPA2 all six-dimensional pools and the 216 pools of single 384-well plates were examined alongside the DNA of *Brassica napus* accession Express, two *Brassica oleracea* accessions, two *Brassica rapa* accessions and the DNA of the four pools of *Brassica* accessions.

### Clone deconvolution

BAC coordinate deconvolution was established in Microsoft Excel (Microsoft Corporation, Redmond, USA). For all 82,944 BAC coordinates the corresponding pool information was entered into an Excel table. All pools that contained a particular SNP were selected with the filter function in order to deduce the list of all putative BAC coordinates for this assay nucleotide.

### Analysis of putative BAC coordinates

The BAC clones which were identified for the different assays were analyzed with the help of gene-specific amplicons (Additional file 4) for the presence of the different *Brassica napus* loci. The validation of the results which resulted from the screening of all six-dimensional pools as well as the SA and SB pools (OPA1) was carried out in two steps. Based on the list of putative BAC coordinates all 384-well plates which contained these clones were selected and DNAs of these pools were used as templates in PCR assays with gene-specific primer pairs in order to identify those plates which contained a particular locus. Only putative BAC clones which were present in these plates were tested individually for the presence of a particular locus by PCR. In cases in which several hundreds or thousands of putative clones were identified it was only analyzed whether the known coordinates for the corresponding loci were among the list of putative clones.

For the assays of OPA2 all putative BAC coordinates which resulted from the screening of the six-dimensional pools and the 216 pools of the individual microtiter plates were screened by PCR with gene-specific amplicons (Additional file 4) for the presence of a particular locus.

All clones that were confirmed to contain loci of interest were included in the list of BAC coordinates known to be present in the library.

## Competing interests

The authors declare that they have no competing interests.

## Authors’ contributions

HXC and RS planned the work. HXC oversaw and participated in the construction of the BAC pools, carried out and evaluated the genotyping, deconvoluted the BAC coordinates, validated the results by PCR and prepared figures. RS conceived the study, contributed to identification and characterization of *Brassica napus* gene sequences, designed gene-specific amplicons, selected the SNP information for the genotyping assays and drafted the manuscript. Both authors read and approved the final draft of the manuscript.

## Supplementary Material

Additional file 1 **Sequence information for oligonucleotide pool assays.** Bold font indicates sequences specific for a particular oligonucleotide class. Illuminocode sequences are shown in italics. Plain font corresponds to sequences of *Brassica napus* alleles and/or the specified genes. Assays with the name affix “A” were included in experiments with oligonucleotide pool assays (OPA) 2 the remainder were part of OPA1. Click here for file

Additional file 2 **Clones detected in the BAC library.** (A) Screening results for SNP assays that discriminated between homoeologous genes in the Express genome. (B) Screening results for SNP assays specific for single genes in the Express genome. (C) Screening results for SNP assays that did not differentiate between homoeologous genes in the Express genome. Click here for file

Additional file 3 **Distribution of normalized Theta values.** Panel (A) shows which proportion of pools that revealed normalized R values of 0.2 or higher were found in the indicated intervals of normalized Theta values. In order to integrate the data of all assays the normalized Theta values were transformed as follows: 0.5-|0.5-normalized Theta|. Grey bars represent data for assays that were specific for single genes in the Express genome (Additional file 2B) and white bars correspond to data for those SNP assays that did not differentiate between homoeologous genes in the Express genome (Additional file 2C). The grey and black bars in panel (B) indicate for each of the assays analyzed which proportion of BAC pools was assigned to the class containing a single SNP (normalized Theta ≤ 0.05 or normalized Theta ≥ 0.95) or both SNPs (0.05 < normalized Theta < 0.95), respectively. Click here for file

Additional file 4 **Gene-specific amplicons.** The table lists all amplicons which were used to analyze the putative BAC clones for the presence of a particular locus by PCR. The oligonucleotides belonging to amplicon BnaA/C.DGAT1.b amplified in *Brassica napus* Express *BnaA*.*DGAT1*.*b* and *BnaC*.*DGAT1*.*b*. Click here for file
