# Supplementary figures and images for: Screening of a Brassica napus bacterial artificial chromosome library using highly parallel single nucleotide polymorphism assays
Source: BMC Genomics. 2013 Sep 6;14:603. doi: 10.1186/1471-2164-14-603 (PMC3846124; doi:10.1186/1471-2164-14-603)

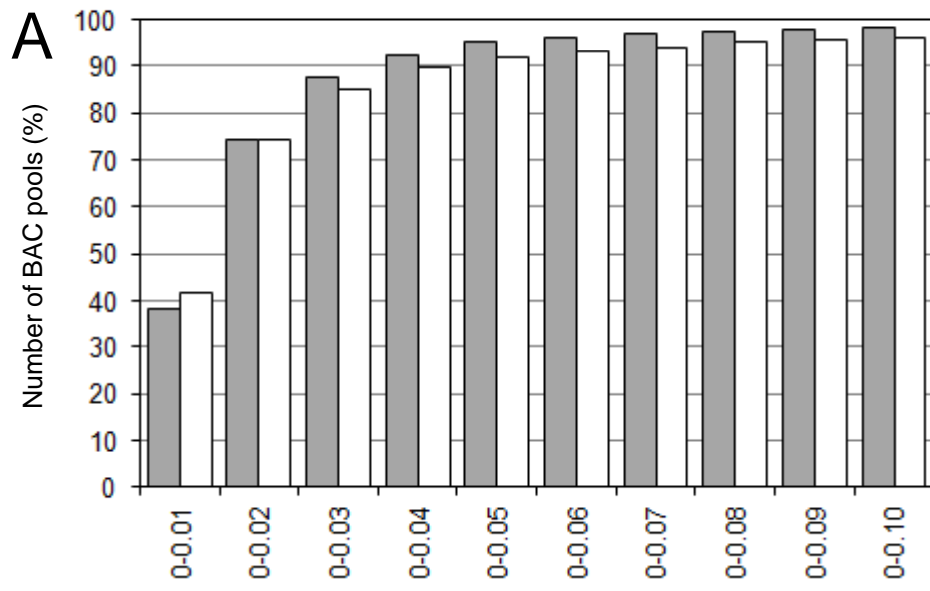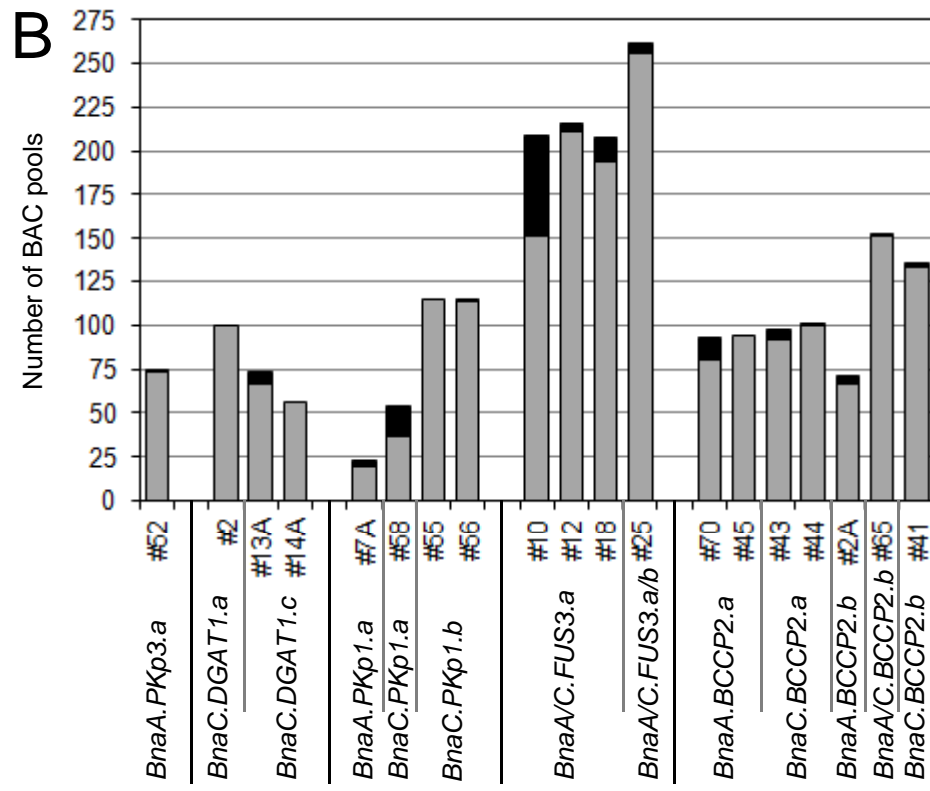

Supplement: Additional file 3 — Distribution of normalized Theta values. Panel (A) shows which proportion of pools that revealed normalized R values of 0.2 or higher were found in the indicated intervals of normalized Theta values. In order to integrate the data of all assays the normalized Theta values were transformed as follows: 0.5-|0.5-normalized Theta|. Grey bars represent data for assays that were specific for single genes in the Express genome (Additional file 2B) and white bars correspond to data for those SNP assays that did not differentiate between homoeologous genes in the Express genome (Additional file 2C). The grey and black bars in panel (B) indicate for each of the assays analyzed which proportion of BAC pools was assigned to the class containing a single SNP (normalized Theta ≤ 0.05 or normalized Theta ≥ 0.95) or both SNPs (0.05 < normalized Theta < 0.95), respectively. [file 1471-2164-14-603-S3.pdf]
